# Thermal, Chemical and pH Induced Denaturation of a Multimeric β-Galactosidase Reveals Multiple Unfolding Pathways

**DOI:** 10.1371/journal.pone.0050380

**Published:** 2012-11-21

**Authors:** Devesh Kishore, Suman Kundu, Arvind M. Kayastha

**Affiliations:** 1 School of Biotechnology, Faculty of Science, Banaras Hindu University, Varanasi, India; 2 Department of Biochemistry, University of Delhi South Campus, New Delhi, India; Russian Academy of Sciences, Institute for Biological Instrumentation, Russian Federation

## Abstract

**Background:**

In this case study, we analysed the properties of unfolded states and pathways leading to complete denaturation of a multimeric chick pea β-galactosidase (*Cp*GAL), as obtained from treatment with guanidium hydrochloride, urea, elevated temperature and extreme pH.

**Methodology/Principal Findings:**

*Cp*GAL, a heterodimeric protein with native molecular mass of 85 kDa, belongs to α+β class of protein. The conformational stability and thermodynamic parameters of *Cp*GAL unfolding in different states were estimated and interpreted using circular dichroism and fluorescence spectroscopic measurements. The enzyme was found to be structurally and functionally stable in the entire pH range and upto 50°C temperature. Further increase in temperature induces unfolding followed by aggregation. Chemical induced denaturation was found to be cooperative and transitions were irreversible, non-coincidental and sigmoidal. Free energy of protein unfolding (ΔG_0_) and unfolding constant (K_obs_) were also calculated for chemically denatured *Cp*GAL.

**Significance:**

The protein seems to use different pathways for unfolding in different environments and is a classical example of how the environment dictates the path a protein might take to fold while its amino acid sequence only defines its final three-dimensional conformation. The knowledge accumulated could be of immense biotechnological significance as well.

## Introduction

β-Galactosidase is an enzyme of industrial importance, mainly characterized by its ability to hydrolyze terminal, non-reducing β-D-galactosyl residues from oligosaccharides and polysaccharides, as well as glycoproteins and glycolipids [1]. Although its physiological importance, regulation of synthesis, enzymatic properties and structural detail from prokaryotes are well-established, little is known about the stability and folding mechanism of β-galactosidase.

A solution to the protein folding enigma is of enormous intellectual importance and might provide the ‘missing link’ in the flow of information from a gene sequence to the 3D structure of a protein. Therefore, exploring the mechanism of protein folding is often referred to as the second half of genetics. The amino acid sequence of a protein predisposes it towards its natural conformation. The folding of protein into its proper 3D conformation is the most fundamental and universal example of biological self assembly; understanding this complex process therefore provide a unique insight into the way in which evolutionary selection has influenced the properties of a molecular system for functional advantage. An appreciation of protein folding will also facilitate far reaching implications, for example in the fine tuning of structure prediction algorithms and the rational design of novel sequences not provided by evolution [2], [3], as well as in the fields ranging from medicine to nanotechnology [4]. In addition to its physiological importance, the frequent use of β-galactosidase as a fusion or marker protein would necessitate creation of variants through protein engineering techniques. Understanding of protein folding mechanism of this enzyme would be precious in such efforts.

The stability of a native protein is a function of surrounding environmental variables such as pH, temperature, ionic strength, and solvent composition as they dislocate various intramolecular bonds, responsible for stability and integration of the protein [5]. In addition, only perfectly folded proteins survive for long term in biologically crowded environment and interact selectively with their natural ligands. Failure to fold into the intended conformation usually produces inactive proteins with fatal properties including toxic prions. Several neurodegenerative diseases, considered as the most menacing of our era, are believed to be an outcome of the accumulation of misfolded proteins in the intra-cytoplasmic regions [6]. For proteins of biotechnological significance like β-galactosidase, understanding of environmental factors that dictate stability and folding will be important for better production of the recombinant enzyme at industrial scale.

An unfolded protein folds along a funnel that describes its free energy until it reaches the native state of minimal free energy level. The multidimensional protein landscape often contains local minima, hosting metastable folding intermediates during thermal or denaturant induced equilibrium unfolding [5], [7], [8]. Proteins are supposed to avoid searching irrelevant conformations and fold rapidly by making local independent decisions first, followed by final global decision [9]. To investigate the folding process, we must understand the microscopic folding routes to the downhill gulley and permissible ways to avoid traps and hills during conformational changes. Such fine understanding will only emerge from investigations of diverse proteins in large numbers. Our investigation of β-galactosidase is a step in this direction and allows us to provide evidence for intermediate states in its unfolding and the multiple pathways that exist in a divergence from classical two state mechanism.

Approximately 75% of the world's population loses the ability to completely digest a physiological dose of lactose after infancy [10]. Therefore, commercial use of β-galactosidase has received widespread interest and applications in food technology, mainly in the dairy industry to improve sweetness, solubility, flavour, and digestibility of dairy products; suitable for lactose-intolerant people [11], [12]. In addition to its biological functions, β-galactosidase has also gained importance as a fusion protein [13], and as a marker or reporter protein [14] for detection of transcriptional activity. Extensive efforts are being made for its economical exploitation by means of mutation and immobilization techniques, aiming at improvement in structural and functional stability of β-galactosidase under broad range of industrial environment. Therefore, understanding structure-function relationship of the enzyme under different environmental conditions is fundamentally important for both theoretical and applicative aspects. Such studies may provide insight into the molecular basis of the stability of the enzyme, which in turn can be used to design protocols and/or a protein with special properties for novel biotechnological applications [5].

## Materials and Methods

Chick pea β-galactosidase (*Cp*GAL) was purified from *Cicer arietinum* as described recently [15]. Homogeneity of purified preparation was checked by SDS-PAGE and size exclusion chromatography (SEC). GdHCl (guanidium hydrochloride), urea, ANS (8-anilino-1-naphthalene sulfonate) and Bradford reagent were procured from Sigma Chemical Co. (St. Louis, MO, USA). All the chemicals for buffers were of analytical grade from Merck (Eurolab GmbH Damstadt, Germany). Milli Q (Millipore, Bedford, MA, USA) water with a resistance of higher than 18 MΩ cm was used all throughout the experiments. Samples for spectroscopic measurement were centrifuged and filtered through 0.45 µM filters, and the exact concentration of the protein and pH were determined before any spectroscopic measurement.

### Circular dichroism (CD) spectroscopy

Circular dichroism (CD) measurements were performed on a pre-calibrated (with 0.1% d-10-camphorsulfonic acid solution) Jasco J-815 CD spectrometer equipped with a constant temperature cell holder. Temperature was controlled and monitored with a water bath (Multitemp; Pharmacia, Sweden). Conformational changes in the secondary structure of protein were monitored in the region between 190 to 250 nm with a protein concentration of 50 µg ml^−1^ (0.60 µmol) in a quartz cuvette (Hellma) with a path length of 1 mm. The scanning speed, band width and data pitch were set to 50 nm min^−1^, 1 nm and 0.5 nm, respectively. Three accumulation of scan were taken (within 600 HT voltage range) and averaged to get the complete spectra. The content of secondary structures (α-helix, β-sheets) of the protein was obtained from K2D2 server by providing the far UV ellipticity data points in the range of 190–240 nm.

### Fluorescence spectroscopy

Fluorescence spectra were collected on Varian Cary eclipse spectrophotometer equipped with Varian Cary temperature controller (peltier multiple holder). Tryptophan was selectively excited at 292 and emission spectra were collected between 310 to 450 nm using Micro fluorometer cell quartz cuvette (Optiglass) with a path length of 10 mm. Scan speed was set to 100 nm min^−1^ using response time of 1 sec. Slit width for both emission and excitation were 10 nm. The protein concentration used for all fluorescence spectra accumulation was 10 µg ml^−1^. All fluorescence spectra were corrected for background intensity as measured with pure buffer.

The extent of exposure of hydrophobic surface in the protein was measured by its ability to bind with the ANS fluorescent dye [16]. A stock solution of ANS was prepared in methanol and the dye concentration was measured using an extinction coefficient (ε) of 5000 M^−1^ cm^−1^ at 350 nm [17]. For estimation of hydrophobic surface of protein, the sample was incubated with a 100 molar fold excess of ANS at room temperature in the dark. The fluorescence was measured after 30 min with excitation at 380 nm and emission between 400 to 600 nm.

### Enzyme activity and protein estimation

The hydrolyzing activity of *Cp*GAL under various conditions of pH, temperature and denaturant was estimated using *o*-nitrophenyl-β-D galactopyranoside (ONPG) substrate, as described recently [15]. Protein content was estimated using Bradford method [18], with crystalline bovine serum albumin as standard protein.

### Stability against pH and temperature

Acid denaturation of β-galactosidase was carried out as a function of pH using glycine-HCl (pH 2.0–3.0), sodium acetate (pH 4.0–5.0), sodium phosphate (pH 6.0–8.0), glycine-NaOH (pH 9.0–10.0) and phosphate-NaOH (pH 11.0–12.0). After addition of protein, the final concentrations of all buffers were adjusted to 20 mM. A stock solution of the protein was added and mixed to the appropriate buffer and was incubated for 18 h at room temperature (25±1°C). Size exclusion chromatography (SEC) was carried out on AKTA FPLC system (GE Healthcare Bio Sciences AB, Uppsala, Sweden) using Superdex 200 10/300 GL column at pH 2.0 and pH 4.0. Fractions of 0.5 mL were collected at a flow rate of 0.5 mL min^−1^.

Temperature-induced denaturation of the enzyme, under given conditions (20 mM acetate buffer, pH 4.0) was performed as a function of increasing temperature using peltier temperature controller. Protein samples were incubated at the desired temperature for 5 min before ellipticity and emission measurements were obtained.

### Chemical induced denaturation

Chemical denaturation of the enzyme was performed with increasing concentrations of the denaturant at pH 4.0. The protein sample was incubated at a desired denaturant concentration for approximately 18 h at room temperature to attain equilibrium.

In all unfolding studies, the extent of denaturation was expressed in terms of fraction unfolded (α_i_) at i^th^ concentration of denaturant and can be calculated from the equation [19]

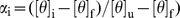
where [θ]_f_ is the ellipticity of the molecule when it is fully folded and [θ]_u_ is the ellipticity of the fully unfolded molecule.

The unfolding constant for a heterodimeric protein was calculated according to the equation given by Greenfield [19]

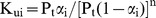
where P_t_ is the molecular concentration of dimeric protein and n is the number of chain formed after denaturation. The free energy of unfolding of a protein at any given concentration of denaturant (ΔG_ui_) can be evaluated in terms of moles of folded protein since one mole of a folded dimeric protein unfolds to give two moles of unfolded chains.
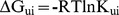
where R is the gas constant (1.98 cal mol^−1^) and T is the absolute temperature. Free energy of protein unfolding (ΔG_0_) was determined from a plot of ΔG_ui_ (y) as a function of denaturant (x) where y intercept equals to the ΔG_0_. A half-Chevron plot was obtained from the values of unfolding kinetics rate constants (K_obs_) plotted against denaturant concentration.

## Results and Discussion

Not much information about molten globule state or other intermediates in multidomain proteins are available. The domains or subunits in native proteins are considered to be independent folding units that assemble and produce native molecules [5]. These structural regions are also expected to unfold independently whether they are isolated or together. Elevated temperatures as well as high concentrations of chemical denaturants dissolve the secondary structure elements and three dimensional integrity of the protein. Therefore, structure-functional relationship and folding behaviour of *Cp*GAL under specific environmental and denaturant condition was studied using circular dichroism, fluorescence spectroscopy and activity measurement.

**Figure 1:**
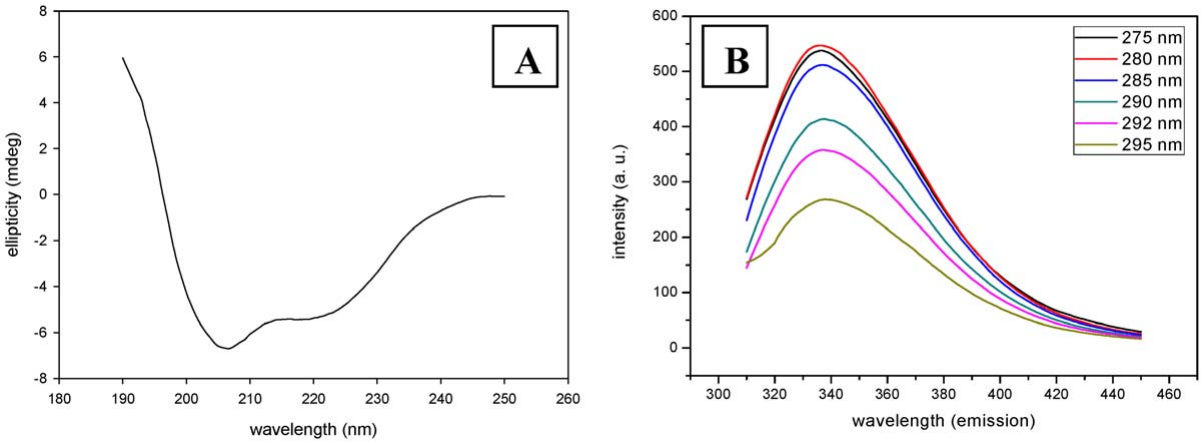
Native spectra for *Cp*GAL. (A) Represents Far UV CD spectra, whereas (B) represents fluorescence emission spectra at different excitation wavelengths.

Chick pea β-galactosidase (*Cp*GAL) is a heterodimeric protein with a native molecular mass of 85 kDa. SDS-PAGE of *Cp*GAL shows two distinct bands corresponding to molecular mass of 48 and 38 kDa [15]. Primary amino acid sequence reveals that it contains 22 tryptophan and 32 tyrosine residues. The enzyme was found to be quite stable in acetate buffer (20 mM, pH 4.0) over weeks as monitored by enzyme activity and SDS-PAGE. Therefore, chemical and thermal denaturation studies were carried out in 20 mM acetate buffer (pH 4.0) [15]. Not many proteins with such distinct acidic characteristics have been investigated likewise. *Cp*GAL belongs to α+β class of protein at pH 4.0, as shown by its characteristic native far UV spectrum measured by circular dichroism (Fig. 1A) [20]. Secondary structural content was determined from the CD spectrum of native protein using K2D2 server, which revealed 30% α-helices and 14% β-sheets at 20 mM acetate buffer (pH 4.0).

Circular dichroism (CD) was used for studying the secondary structure and conformational changes that occurred during unfolding of the protein. The wavelengths and magnitudes of the ellipticity bands usually show complete dependence on structural conformation of protein, making it a useful index of protein unfolding. Protein with a high degree of conformation showed distinctive CD spectra, which vanished gradually during unfolding of proteins [21].

Fluorescence spectra provide additional sensitive means of characterizing proteins and their conformation. The spectrum is determined chiefly by polarity of the environment of the tryptophan and tyrosine residues and by their specific interactions [8]. Basically, the fluorescence emission maximum suffers a red shift when chromophores become more exposed to solvent and the quantum yield of fluorescence decreases when the chromophores interact with quenching agents, either in a solvent or in the protein itself. To determine the intrinsic fluorescence properties, emission spectra were obtained at varying excitation wavelengths (Fig. 1B). The spectra showed similar emission maximum (λ_max_) with varying intensity at different excitation wavelengths. Both tyrosine and tryptophan residues get excited at lower wavelength (275–285 nm), and emit with higher intensity; however, only tryptophan gets excited beyond 290 nm and emit at lower intensity with similar λ_max_ emission. Therefore, tryptophan was selectively excited at 292 nm wavelength for all fluorescence experiments as its fluorescence is strongly influenced by the proximity of other nearby protonated groups such as Asp or Glu.

Due to high sensitivity of tryptophan fluorescence with respect to changes in the microenvironment of the intrinsic chromophores, various properties of the unfolded state can have a distinct effect on intensity values. In particular, differences in the extent of protein aggregation, in structural compactness, or simply in the structural arrangement of tryptophan residues in unfolded states can significantly change the magnitude and intensity of the red shift observed during the unfolding transition [8].

### Thermal denaturation

**Figure 2:**
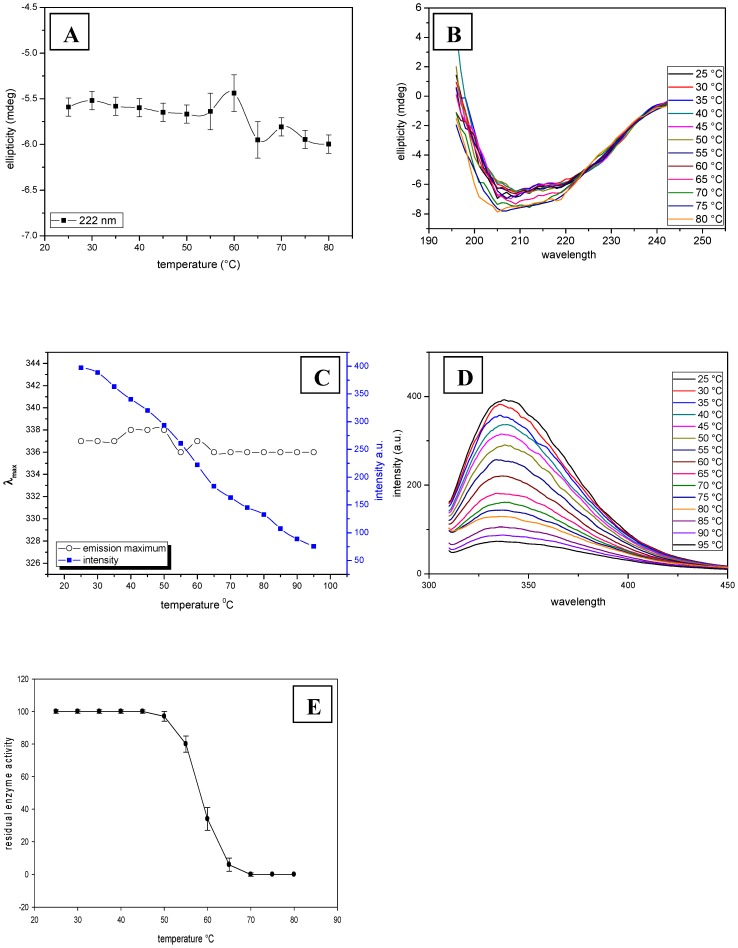
Thermal denaturation. Ellipticity (A & B) and emission (C & D) measurement of *Cp*GAL as a function of temperature. Emission shows monotonous decrease in the intensity of λ_max_ with increasing temperature. (E) Represents residual enzymatic activity as a function of heat denaturation for 5 min, at respective temperatures.

Most proteins are characterized by well-defined three-dimensional structures, which in general exist only within the limits of specific environmental conditions. Outside these conditions, proteins exhibit denatured and structurally unfolded states. The enzymatic activity of *Cp*GAL was found to be unperturbed upto 50°C (Fig. 2E). The enzyme loses 68% residual enzyme activity within 5 min at 60°C, whereas complete loss in enzyme activity was seen around 65°C. However, *Cp*GAL did not show any significant changes in ellipticity (θ_222_) and emission maximum (λ_max_) measurement with respect to increasing temperature (Fig. 2A–E). Thermally denatured *Cp*GAL did not restored maximal fluorescence intensity or enzymatic activity even after prolonged cooling. These results suggest that the enzyme is structurally and functionally very stable upto 50°C. With further increase in temperature, enzyme probably undergoes denaturation with concomitant aggregation. Presence of several tryptophan residues in the primary amino acid sequence also suggests that the protein is highly vulnerable to aggregation under elevated temperature. In addition, proper thermal unfolding of the enzyme must have completely vanished the characteristic ellipticity along with corresponding red-shift in emission maximum, as were seen under chemical denaturation.

In general, the thermal unfolding transitions of multidomain proteins are accompanied by irreversible aggregation at elevated temperature [8], [22], [23], [24], [25]. As a consequence, the analysis of thermostabilities in terms of equilibrium thermodynamics is not applicable if the irreversible process is fast with respect to the structural unfolding transition [22], [26], [27]. In addition, due to the fact that aggregation is related to an interaction of at least two molecules, the process strongly depends on the protein concentration as well.

The circular dichroism might not show an ellipticity decrease due to scattering from aggregates, which can bias the ellipticity. Furthermore, fluorescence did not show any statistically significant change in tryptophan emission maximum (λ_max_); however the intensity (I_max_) decreased linearly as a function of temperature (Fig. 2C & D). Due to aggregation, the tryptophan residues could not properly expose to the solvent and remains buried, as reflected by unaltered emission maximum. The reduction in fluorescence intensity could be attributed to the decrease in distance between tryptophan and specific quenching residues such as protonated carboxyl, protonated imidazole, deprotonated ε-amino groups and tyrosinate, which quench tryptophan residues effectively at elevated temperature [28]. A monotonic decrease in intensity with increasing temperature also indicates that heating induces a conformational change because the polarity of the regions, to which the tryptophans are being exposed, must be changing [8], [29]. Similar type of behaviour due to aggregation has been shown by thermal denaturation of α-amylases [8], [22].

This is the first report of thermal denaturation of any β-galacosidase, and further studies are needed to get additional insight of thermal denaturation kinetics.

### pH denaturation

**Figure 3:**
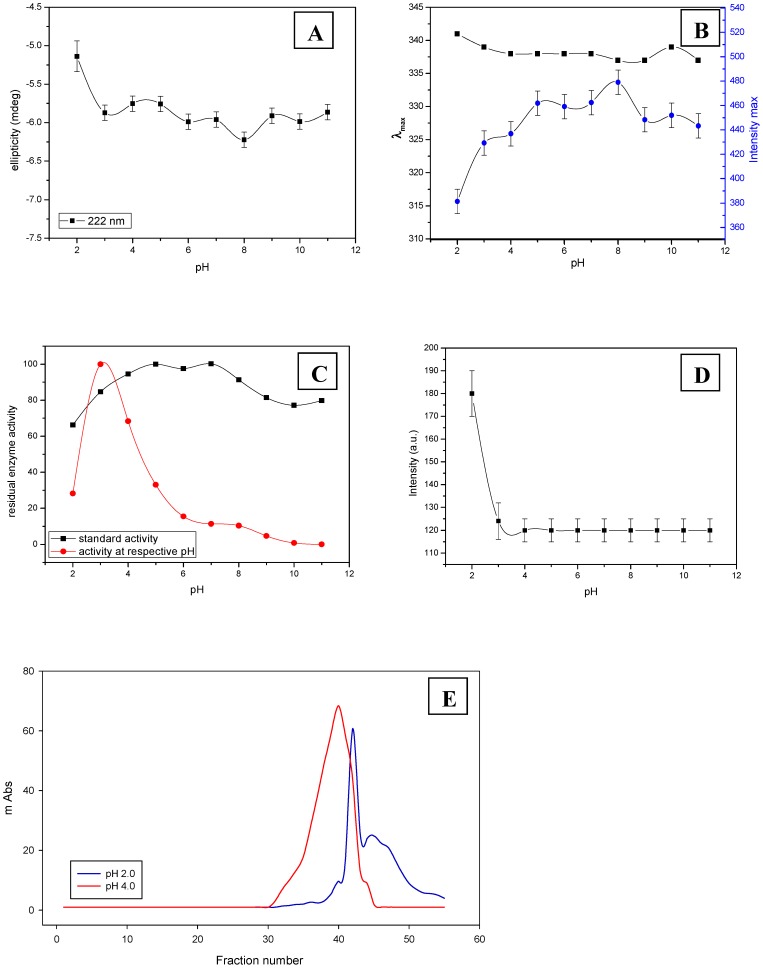
pH induced denaturation. Ellipticity (A), emission (B) and enzymatic activity (C) measurement of *Cp*GAL as a function of pH. The enzymatic activity was estimated at respective pH of incubation and at pH 2.8 (according to the standard activity assay protocol). (D) Represents fluorescence intensity of ANS binding. (E) Represents size exclusion chromatography (SEC) profile of *Cp*GAL at pH 2.0 and pH 4.0.

At extreme pH, the main force unfolding the protein is the repulsion between charged groups on the protein molecule. *Cp*GAL exhibits structural and functional stability over a wide range of pH. CD and fluorescence spectroscopy did not show significant structural changes in the range of pH 3.0–11.0 with minor changes at pH 2.0 (Fig. 3A & B). Enzymatic activity was estimated according to the standard assay procedure (at optimum pH 2.8) and at their respective pH of incubation. Enzyme activity estimated at different pH revealed that the activity peaks sharply around pH 3.0 and rapidly decreased in either direction [15]. However, when an aliquot was withdrawn from enzyme incubated with different pH, and added to the standard reaction mixture (pH 2.8), more than 80% enzymatic activity was recovered for all samples except for the one incubated at pH 2.0 (Fig. 3C). Enzyme incubated between pH 4.0–8.0 exhibited more than 92% recovery of enzymatic activity, indicating that the enzyme is structurally and functionally very stable in the pH range of 4.0–8.0.

With further reduction of the pH to 2.0, protein showed disruption of tertiary structure with irreversible loss of about 35% residual enzymatic activity. A small shift in both emission maximum and ellipticity was also observed at pH 2.0. Emission maximum increased from 337 nm to 341 nm, whereas I_max_ dropped from 414 to 374. A shift of 0.61 mdeg in ellipticity (θ_222_) was also observed at pH 2.0. The exposure of any hydrophobic regions, buried inside the enzyme in the native state, on acid unfolding, was also monitored by ANS binding to the protein. ANS binding did not show any significant change in fluorescence intensity in the range of pH 3.0–11.0, but increased about 1.5 fold at pH 2.0 (Fig. 3D), whereas emission showed a blue shift of about 14 nm. Thus it seems that at extreme pH, the enzyme becomes less compact than the native molecule, and more hydrophobic binding sites are accessible to ANS. The dissociation of the dimers into monomers, however, is also a distinct possibility and was verified by size exclusion chromatography at low pH. Size exclusion chromatography of *Cp*GAL indicates that the protein resolves into two peaks of lower molecular mass at pH 2.0 (Fig. 3E). In addition, the resolved peaks at pH 2.0 completely lost the enzymatic activity. The enzyme seems to be very fragile at pH 2.0, as it regains only 65% enzymatic activity even after 18 h of incubation with minute shifts in ellipticity and fluorescence. Probably the interaction between two subunits of heterodimeric conformation falls to minimum at this pH and gets separated during size exclusion chromatography.

pH denatured state is thus quite different from thermal denatured state. In contrast to thermal denaturation, the conformation of *Cp*GAL shows structural relaxation at pH 2.0, with decreased θ_222_, I_max_ and red shift in λ_max_. In addition, ANS binding also showed appearance of more accessible hydrophobic residues, and hence a less compact conformation of protein compared to native state. Both secondary and tertiary structure undergoes perturbation along with enzymatic activity, which represents a partial denatured intermediate state of protein at pH 2.0 where the subunits are dissociated. In contrast to other samples subjected to pH treatment, the sample at pH 2.0 showed an irreversible loss of about 35% enzyme activity following restitution at optimum pH of storage (pH 2.8), and hence manifests the existence of a partially reversible intermediate state. Such characteristics were not observed during thermal denaturation, indicating lower probability of monomer formation in measurable amounts during thermal denaturation.

The above observation, combined with the fact that the secondary and tertiary structural content is largely intact, showed that the enzyme is mostly stable in the pH range 2.0–11.0. It does not lose its catalytic activity even after prolonged exposure to the pH range 3.0–11.0, and enzymatic activity recovers fairly well, when the enzyme reverts back to the acidic pH 3.0–4.0. In addition, the unfolded state at pH 2.0, exhibiting a reduced secondary structure and activity, represents the acid-unfolded state of the enzyme [30], indicating a partial unfolding of the protein molecule.

Pea β-galactosidase has shown structural switching from α/β to α+β, following a change in pH 5.0 to pH 8.0 [31]. However, *Cp*GAL remains in α+β conformation all throughout the pH range (2.0–11.0) as manifested by far UV CD spectroscopy.

### Chemical denaturation

The protein was denatured chemically with different concentration of GdHCl or urea at pH 4 for 18 h, prior to spectral measurements.

**Figure 4:**
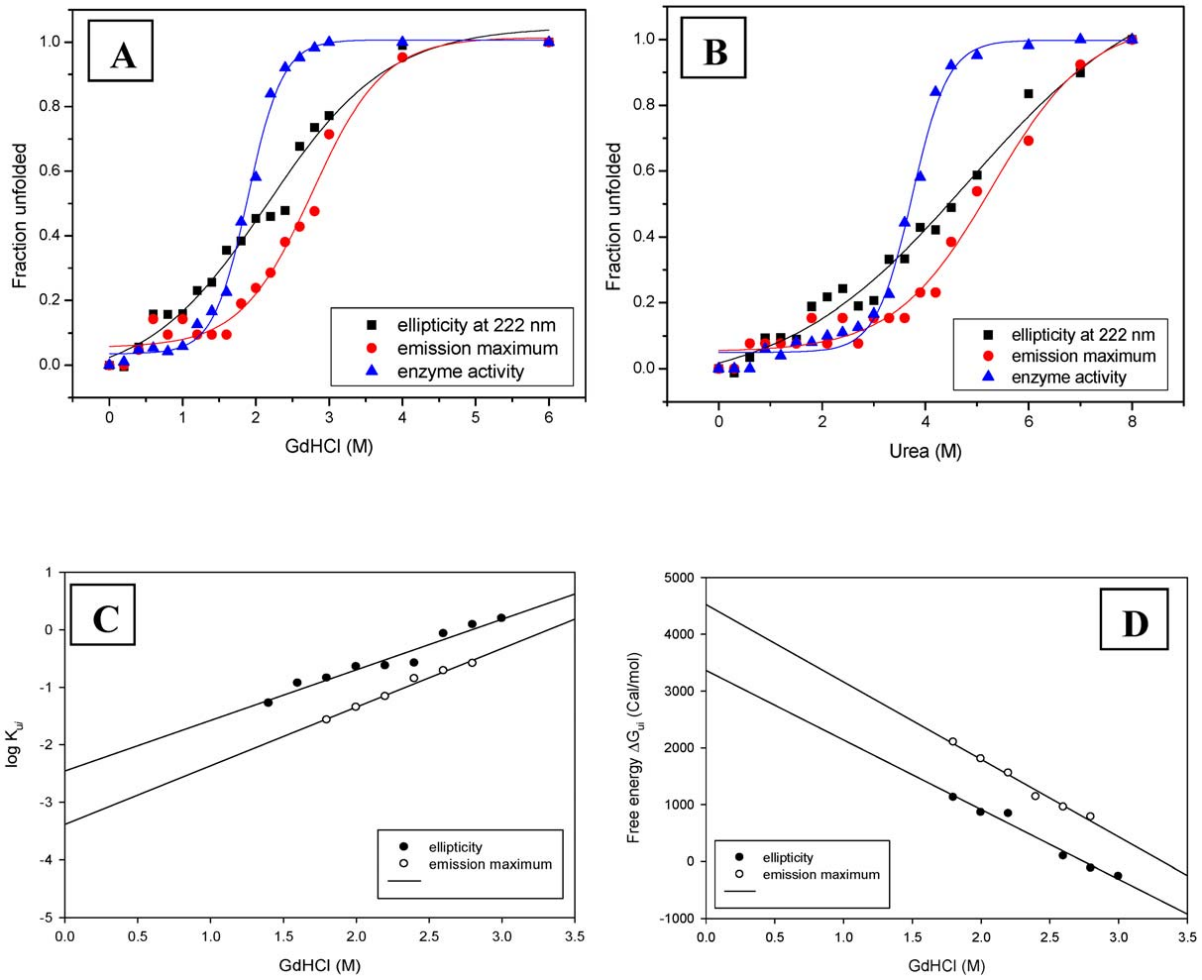
Chemical induced conformational changes. (A) & (B) represents GdHCl and urea mediated transitions of protein unfolding, respectively. (C) Represents free energy of unfolding, whereas (D) represents unfolding kinetics rate constants (Half Chevron plot) against GdHCl. Intercepts at y axis gives ΔG_0_ and log K_obs_, respectively.

GdHCl induced denaturation showed cooperative transitions that are irreversible, non-coincidental and sigmoidal (Fig. 4A). The ellipticity decreases below the concentration of 1 M GdHCl concentration, while fluorescence and enzymatic activity does not change significantly up to this point. Interestingly, the melting of secondary structure takes place earlier to active site perturbation and microenvironment changes of tryptophan. Pea β-galactosidase showed similar GdHCl denaturation pathway, where secondary structure falls ahead of tertiary conformation and enzymatic activity [31]. *E coli* β-galactosidase also showed similar pattern of urea induced denaturation [1]. Pea β-galactosidase follows simple two states unfolding under GdHCl induced denaturation at pH 8.0. However when the denaturation was studied at pH 5.0, it followed four state unfolding pattern with presence of two molten globule intermediates [31]. The non-coincidental transitions for *Cp*GAL do indicate the presence of intermediates, which however, are not directly evident in GdHCl induced unfolding pathway and might be transient disallowing their accumulation.

Urea does not unfold *Cp*GAL at lower concentrations. However, similar to GdHCl induced unfolding, the ellipticity showed decline before any change could be observed in emission maximum and activity. Urea completely denatured the protein at 8 M concentration as shown by enzymatic activity, fluorescence and ellipticity; however, activity and emission maximum does not change up to 2.5 M urea concentration (Fig. 4B). Urea mediated unfolding was also found to be cooperative and transitions were irreversible, non-coincidental and sigmoidal. Such non-coincidental transition indicates the probable existence of an intermediate state in unfolding [32]. *E coli* β-galactosidase was shown to be composed of five compact domains [33] that underwent completely reversible urea denaturation kinetics with midpoints of transition at 3.655 M urea. The unfolding of the enzyme followed four states model, including two partially folded dimeric intermediates. Denatured β-galactosidase refolds only in the presence of 0.8–1.6 M urea concentration; to avoid the aggregation of the side chain, which were practically minimized under these conditions. The folding mechanism of chemically denatured *E. coli* β-galactosidase revealed three stages. It starts with the formation of secondary structure, followed by collapse to subdomains and monomers and finally associates to form the native quaternary structure [1]. The refolded enzyme was found to be indistinguishable from native enzyme, in terms of biochemical and biophysical characteristics [1].

The equilibrium unfolding of *Cp*GAL by various denaturants revealed a high stability of the enzyme. *Cp*GAL retains structural integrity and enzymatic activity in the presence of 1.4 M GdHCl as well as in the presence of 2.5 M urea, indicating the high rigidity of the molecule. A red shift of 18 nm (337 nm to 355 nm) in the wavelength emission maximum of intrinsic fluorescence was observed upon complete chemical induced unfolding. The observed red shift is caused by the fact that tryptophan residues are replaced from the less polar interior of the protein to solvent exposed regions upon unfolding. The shape of the emission spectrum of pure L-tryptophan coincides best with the spectrum of the urea or GdHCl induced unfolded state of *Cp*GAL, indicating that this state represents more or less fully solvent accessible tryptophan residues. Chemical denaturation thus completely unfolds the protein into its inactive, monomeric linear forms unlike pH or thermal denaturation.

The C_m_ value for enzymatic activity was found to be 1.87 and 3.72 M for GdHCl and urea, respectively. The free energy of protein unfolding (ΔG_0_) and rate constant of chemical induced unfolding (K_obs_) was also calculated as given in the methods section (Fig. 4C & D). Half Chevron plot was constructed from the values of unfolding kinetics rate constants (K_ui_) plotted against denaturant concentration, where rate constant of unfolding (K_obs_) equals the value of the y intercept. The transition midpoints by far UV CD and fluorescence emission are summarized in Table 1.

**Table 1:** Unfolding parameters of chemically denatured *Cp*GAL.

Kinetics parameter	GdHCl denaturation		Urea denaturation	
	Far UV CD	Fluorescence	Far UV CD	Fluorescence
**ΔG_0_ (Kcal/mol)**	3.35	4.50	3.79	5.46
**Log K_obs_**	−2.47	−3.38	−2.85	−4.12
**C_m_ (M)**	2.15	2.70	4.46	5.05

The C_m_ value for GdHCl denatured pea β-galactosidase was observed in the range of 1.5–2.0 M at pH 8.0, whereas it was found to be around 2.5 M at pH 5.0, when measured using θ_222_, λ_max_ and enzymatic activity [31]. The free energy at pH 5.0 was calculated to be 11.03, 4.88 and 5.26 kcal mol^−1^, corresponding to θ_222_, λ_max_ and enzymatic activity, respectively. The analogous values at pH 8.0 were 4.14, 2.07 and 1.89 kcal mol^−1^, respectively. The C_m_ value for urea denatured *E. coli* β-galactosidase was found to be in the range of 3.5 to 3.8 M urea, as obtained from λ_max_, θ_222_ and enzymatic activity measurement [1]. These values are in order with those observed for *Cp*GAL.

## Conclusions

**Figure 5:**
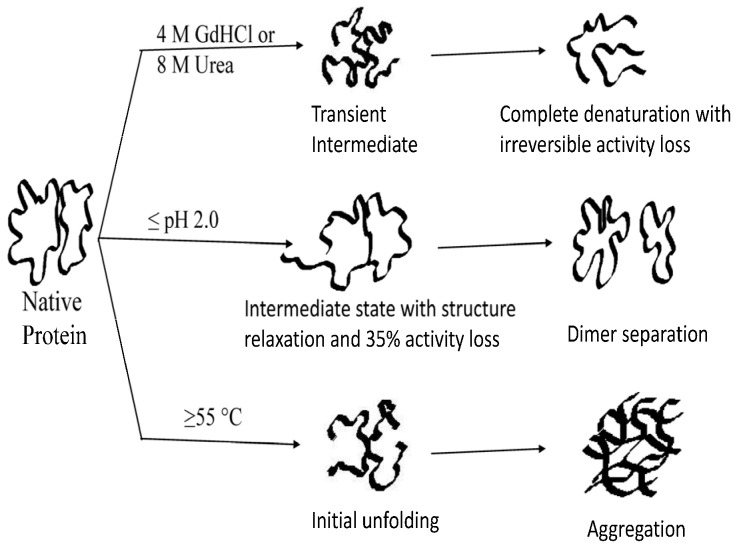
Proposed denaturation scheme for *Cp*GAL. The enzyme uses different pathways of unfolding for different denaturants.

Exploring the structure and dynamics of intermediates of folding pathway is necessary not only to understand the mechanism of the protein folding but also to shed light on many natural or disease-related processes and biotechnological applications. Therefore, unfolding behaviour and kinetics was studied for *Cp*GAL, as a model for heterodimeric protein (Fig. 5).

In the far UV region, native *Cp*GAL revealed two well-resolved negative peaks at 208 and 222 nm. The signals at 208 nm were more prominent, indicating high level of structural integrity of the protein. The enzyme loses all of its secondary structural integrity around 4 M GdHCl or 8 M urea, as is evident by complete disappearance of all the characteristic peaks in far UV spectra. When excited at 292 nm, the enzyme showed fluorescence emission maximum (λ_max_) at 337 nm in the native state; whereas, it shifts to 355 nm with 40–45% drop in intensity (I_max_) under complete chemical denaturation conditions. Similar shifts in λ_max_ and I_max_ for various proteins under chemical induced denaturation has been reported previously [5], [7], [34], [35]. The stability of pea β-galactosidase was shown to be dependent on protein concentration. The enhanced protein concentration (≥100 µg ml^−1^) leads to its multimerization, which plays a key role in stabilization, retention of tertiary structure and enzyme activity [31]. In contrast, stability of *Cp*GAL was found to be almost independent of protein concentration, when checked in the range of 10 µg ml^−1^–2 mg ml^−1^; up to a week [15]. As for industrial applications, protein concentration independence in the stability could be a distinct advantage for *Cp*GAL over other galactosidases.

In principle, unfolded states are rather heterogeneous and the mechanisms leading to unfolded states are rather different for thermal, pH or GdHCl induced unfolding [29]. Therefore, the respective unfolded states can have rather different structures. For example, more compact structures of the acidic unfolded states as compared to chemical unfolded states would also explain more buried tryptophan residues which exhibit smaller red shifts. Furthermore, the conformational scrambling of both unfolded states might be different due to different mechanisms of denaturing the proteins. It is obvious that this also may have different effects on the microenvironment of tryptophan residues. Such general observations were also found to be true for the unfolding process of heterodimeric *Cp*GAL. In addition, although the enzyme diverged from the classical two-state mechanism of unfolding, it conforms to many of the established rules and theories for folding like the adherence to protein folding funnel energy landscape. Since many of these theories were built on experimental observations with small, monomeric proteins, the current investigation justifies the fact that many of these theories will hold true for larger, heterooligomeric proteins as well.

*Cp*GAL demonstrates environment dependent variation in unfolding pathways (Fig. 5). Chemical induced denaturation leads to complete unfolding of the enzyme and separation of monomers through transient intermediates which do not accumulate in measurable yields, but is evident from non-coincidental and often broad transition curves (Fig. 4). pH induced denaturation leads to the monomeric dissociation with an intermediate state, characterized by structural relaxation. The dissociated monomers can finally unfold. Thermal treatment initiates denaturation at 55°C followed by immediate aggregation, which could not be monitored by ellipticity and emission. The thermal investigation previously not reported for galactosidases, however, opens up a new dimension for research with the possibility of obtaining new insight in similar classes of enzymes.

This is a classical case proving that while amino acid sequence determines the final native conformation of a protein, the environment dictates the pathway that a protein would take to fold and attain the three-dimensional conformation required for its physiological conformation. This probably allows the protein to fold and function in even challenging environment that may differ from its normal environment under certain circumstances. Such ability might have also helped proteins to survive evolutionary selection pressure and even to attain environment specific conformations since we now know that most proteins acquire closely similar conformations instead of a unique conformation. Apart from such academic insight, the investigation also reveals that one needs to be careful about the processing methods for recombinant galactosidases for biotechnological or industrial purposes.
